# Supplementary material for: Characterizing cell-type spatial relationships across length scales in spatially resolved omics data
Source: Nat Commun. 2025 Jan 3;16:350. doi: 10.1038/s41467-024-55700-1 (PMC11699133; doi:10.1038/s41467-024-55700-1)
Supplement: Supplementary file 3 — Supplementary Software 1 [file 41467_2024_55700_MOESM3_ESM.zip › CRAWDAD/plots/supplementary/S7_cluster_exp_KSFB_annot.pdf]

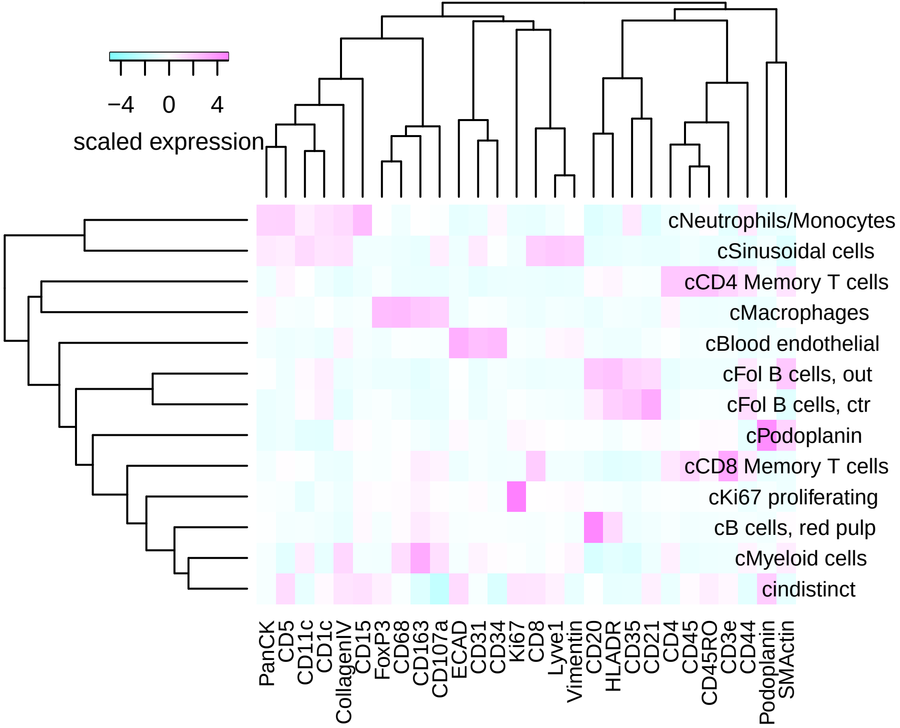

−4
0
4
scaled expression
cNeutrophils/Monocytes
cSinusoidal cells
cCD4 Memory T cells
cMacrophages
cBlood endothelial
cFol B cells, out
cFol B cells, ctr
cPodoplanin
cCD8 Memory T cells
cKi67 proliferating
cB cells, red pulp
cMyeloid cells
cindistinct
PanCK
CD5
CD11c
CD1c
CollagenIV
CD15
FoxP3
CD68
CD163
CD107a
ECAD
CD31
CD34
Ki67
CD8
Lyve1
Vimentin
CD20
HLADR
CD35
CD21
CD4
CD45
CD45RO
CD3e
CD44
Podoplanin
SMActin
